# Analyzing Mass Spectrometry Imaging Data of ^13^C-Labeled Phospholipids in *Camelina sativa* and *Thlaspi arvense* (Pennycress) Embryos

**DOI:** 10.3390/metabo11030148

**Published:** 2021-03-04

**Authors:** Trevor B. Romsdahl, Shrikaar Kambhampati, Somnath Koley, Umesh P. Yadav, Ana Paula Alonso, Doug K. Allen, Kent D. Chapman

**Affiliations:** 1 Department of Biological Sciences & BioDiscovery Institute, University of North Texas, Denton, TX 76203, USA; trevor.romsdahl@unt.edu (T.B.R.); umeshprasad.yadav@unt.edu (U.P.Y.); Anapaula.Alonso@unt.edu (A.P.A.); 2 Donald Danforth Plant Science Center, St. Louis, MO 63132, USA; SKambhampati@danforthcenter.org (S.K.); SKoley@danforthcenter.org (S.K.); 3 United States Department of Agriculture, Agriculture Research Service, St. Louis, MO 63132, USA

**Keywords:** mass spectrometry imaging, MALDI (matrix assisted laser desorption/ionization), metabolomics, lipidomics, ^13^C-labeling, oilseed

## Abstract

The combination of ^13^C-isotopic labeling and mass spectrometry imaging (MSI) offers an approach to analyze metabolic flux in situ. However, combining isotopic labeling and MSI presents technical challenges ranging from sample preparation, label incorporation, data collection, and analysis. Isotopic labeling and MSI individually create large, complex data sets, and this is compounded when both methods are combined. Therefore, analyzing isotopically labeled MSI data requires streamlined procedures to support biologically meaningful interpretations. Using currently available software and techniques, here we describe a workflow to analyze ^13^C-labeled isotopologues of the membrane lipid and storage oil lipid intermediate―phosphatidylcholine (PC). Our results with embryos of the oilseed crops, *Camelina sativa* and *Thlaspi arvense* (pennycress), demonstrated greater ^13^C-isotopic labeling in the cotyledons of developing embryos compared with the embryonic axis. Greater isotopic enrichment in PC molecular species with more saturated and longer chain fatty acids suggest different flux patterns related to fatty acid desaturation and elongation pathways. The ability to evaluate MSI data of isotopically labeled plant embryos will facilitate the potential to investigate spatial aspects of metabolic flux in situ.

## 1. Introduction

Mass spectrometry imaging (MSI) is an analytical technique that maps the spatial distributions of metabolites in tissues using a mass spectrometer that can collect ions along the plane of a tissue surface or cross-section [1]. One of the most common methods of MSI is through matrix assisted laser desorption/ionization (MALDI), where a laser is rastered across a tissue surface to ionize analytes. Ions produced by the laser are detected by mass-to-charge ratio (*m/z*), resulting in metabolite abundances at each location. To visualize the distribution of metabolites, the *m/z* value and its relative intensity are collected at each laser spot and then plotted within a XY-coordinate spatial arrangement. Metabolite signals are represented as pixels and converted to a color scheme to visualize relative abundance [2].

MSI is a powerful technique for maintaining intact spatial-level information (e.g., differences in tissues) that would otherwise be lost in the process during tissue homogenization and extraction. However, MSI has some limitations. MSI is not linked to a chromatogram and therefore individual metabolites are not separated. Since each laser spot will contain a complex mixture of analytes in varying abundances with differences in ionization, MSI can suffer from ion suppression effects [3]. Though MSI can be performed on a high-resolution instrument to distinguish chemical compositions, species that are isobaric, or nearly so, will require additional validation experiments to support compound identification and relative quantification [4]. Finally, MSI captures a snapshot of metabolism at the time of sampling without defining rates of production and turnover of intermediates. To define fluxes quantitatively would require multiple time points from an isotopic labeling experiment and could result in an overwhelming amount of data to analyze. Thus, MSI typically provides an inventory of metabolite locations at a specific time along with relative abundances, but it is limited in its ability to describe metabolite changes within a metabolic network.

In contrast, steady state isotopic labeling experiments can be used to assess the flux through a metabolic pathway or network. When steady state isotopic labeling experiments are leveraged with inspection of many metabolites, the flux analysis has sometimes been described as ‘fluxomics’ [5]. Common isotopes for flux analysis include ^13^C, ^2^H, and ^15^N [6]. These isotopically enriched compounds are supplied to cells or whole organisms so that the heavier isotopes within the labeled substrates are converted into the endogenous metabolites, creating pools of metabolites that are mixtures of isotopes [7]. For example, developing plant embryos may be labeled with ^13^C-labeled sugars, which enter metabolism at glycolysis, or with ^13^C-labeled amino acids, which may be incorporated into the TCA cycle [8,9,10,11,12,13,14]. Isotopically enriched metabolites are distinguishable due to the incorporation of a heavy isotope and can be quantified via mass spectrometry techniques. The relative amounts of labeled versus unlabeled metabolites, and the extent of labeling within metabolites, helps to determine the rate of reactions and the fluxes within a given metabolic network [7].

However, labeled samples are usually harvested via extraction-based methods requiring tissue disruption which results in a loss of spatial metabolic organization, and thus may mask cell-type- or tissue-specific differences in metabolism. Recognizing that isotopic signatures in metabolites reflect the pathways of use, methods to resolve subcellular or some cellular features to guide flux analysis have been established [3,4,15]. Combining MSI and steady state labeling approaches can provide a comprehensive description of metabolism capable of distinguishing activities in different cell types or heterogeneity within tissues. MSI measures metabolites in situ, as they are distributed in tissues. Careful sectioning for MSI limits mixing of cellular contents between tissues so that the in vivo conditions are upheld and labeling can provide information about the flow of carbon.

Plant oilseed embryos are particularly amenable to both MSI and steady state labeling, and have been investigated by each of these techniques separately. Embryos of oilseeds direct significant carbon through primary metabolism toward the biosynthesis of storage lipids. Due to the importance of vegetable oils to human nutrition and biotechnological applications, lipid metabolism in oilseeds, including emerging oilseed crops such as *Camelina sativa* and *Thlaspi arvense*, have received considerable attention [16,17,18,19,20,21]. Lipid metabolites are particularly well suited to ionization and analysis by MALDI-MS. The imaging of distinct tissue types in seeds (e.g., cotyledons and embryonic axis) has revealed unexpected heterogeneous lipid distributions [22,23,24,25,26,27,28]. Developing embryos of oilseeds typically accumulate storage lipids during development, up to 30% or more, and are particularly suited to this spatial analysis. In addition, seeds also exhibit extended durations of metabolic steady state and readily incorporate exogenous isotopically labeled substrates into storage lipids [15]. However, the combination of isotopic labeling with MSI will result in large and complex data sets of numerous isotopologues that require a systematic approach toward analysis. Here, as an example of combining these two approaches together, we demonstrate analysis of ^13^C-MSI data for the molecular species of the membrane lipid and storage lipid intermediate phosphatidylcholine (PC) from ^13^C-labeled *Camelina sativa* and *Thlaspi arvense* (pennycress) embryos; developing a workflow that can be expanded for future ^13^C-MSI studies.

## 2. Results and Discussion

### 2.1. Research Questions Addressed with ^13^C-MSI

**Figure 1:**
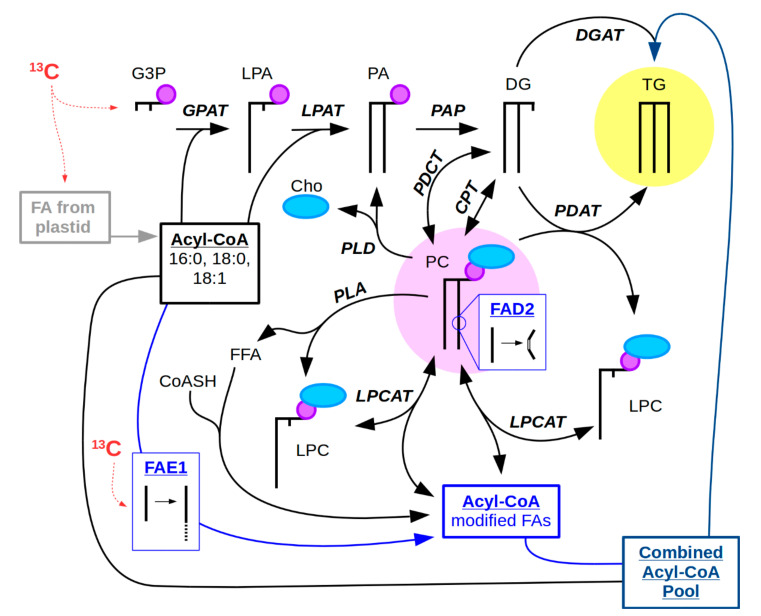
Metabolic pathway of PC and TG synthesis. (Abbreviations: Metabolites: Cho, choline; CoASH, coenzyme A; DG, diacylglycerol; FFA, free fatty acid; G3P, glycerol-3-phosphate; LPA, lysophosphatidic acid; LPC, lysophosphatidylcholine; PA, phosphatidic acid; PC, phosphatidylcholine; TG, triacylglycerol. Enzymes: CPT, choline phosphotransferase; DGAT, DG acyltransferase; FAD2, fatty acid desaturase 2; FAE1, fatty acid elongase 1; GPAT, G3P acyltransferase; LPAT, LPA acyltransferase; LPCAT, LPC:PC acyltransferase; PAP, PA phosphatase; PDAT, phospholipid:DG acyltransferase; PDCT, PC:DG phosphocholine transferase; PLA, phospholipase A; PLD, phospholipase D.) PC (circled in pink) stands as a central metabolite in the synthesis of seed storage oil, a majority of which is TG (circled in yellow), but is also an intermediate for FA modification by desaturation, such as by FAD2. Another type of FA modification includes the elongation of FAs by FAE1 on acyl-CoA substrates. The possible routes of ^13^C-isotope incorporation (red) can occur on the glycerol backbone, from FA synthesis that occurs in the plastid of plant embryos, or from the elongation of FAs by FAE1 in the cytosol. Thick black lines represent FAs and/or FAs bound to glycerol, small purple circle represents phosphate, and light blue oval represents choline.

Here we refer to the combination of MS imaging with steady state isotopic labeling using ^13^C-labeled substrates as ^13^C-MSI. While fluxomics using steady state labeling is focused on answering questions regarding the rates of metabolite interconversion within a cell, MSI addresses spatial distinction of metabolic processes at a tissue/cellular level. In plant embryos, storage lipids provide an energy reserve for use during seed germination. In oilseeds, storage lipids are predominantly in the form of triacylglycerols (TG) packaged into lipid droplets [29]. TG is synthesized by a variety of metabolic routes. The better known route is through the Kennedy pathway, which involves the sequential acylation of a glycerol backbone beginning with glycerol-3-phosphate (Figure 1) [30]. Alternatively, TG can also be synthesized through an acyl-CoA independent pathway involving transacylation of PC to diacylglycerol (DG) [31]. The fatty acids (FAs) synthesized from plastids and used in acylation of the glycerol backbone are long chain FAs of 16 and 18 carbon in length, and are either saturated or monounsaturated when they are exported from the plastid. However, FAs can be further modified in the endoplasmic reticulum (ER) by additional desaturation or elongation. Desaturation of FAs outside of the chloroplast occurs on a PC substrate, e.g., by FAD2 (Figure 1) [32]. Desaturated FAs can then be cleaved from PC to re-enter the acyl-CoA pool by LPCAT (lysophosphatidylcholine acyltransferase) and used by various acyltransferases. Alternatively, the desaturated PC can be used to directly acylate DG with PDAT (PC:DG acyltransferase) to produce TG [31,33]. By contrast to desaturation, elongation of FAs by fatty acid elongase 1 (FAE1) uses acyl-CoA substrates (Figure 1) [34]. Elongated FAs can then be used to acylate the glycerol backbone of DG to form TG by DGAT (DG acyltransferase) or re-enter PC through the acyl editing cycle (Figure 1) [35,36]. Thus, FA modification can take multiple routes, and PC is an important intermediate for TG synthesis in addition to its role as a major structural lipid in membranes. PC is utilized for TG synthesis through a number of mechanisms including transacylation of DG, interconversion of PC and DG by PDCT and CPT, or through generation of the DG precursor, phosphatidic acid (PA), by phospholipase D (Figure 1). Given this central importance of PC in TG synthesis in developing embryos, we sought to visualize different PC molecular species using ^13^C-MSI to assess potential spatial differences in carbon flux through PC.

### 2.2. Considerations for ^13^C-Labeling in ^13^C-MSI Experiments

An important consideration for isotopic labeling is that the provision of the heavy isotope does not perturb metabolism. Thus, both the introduction of the isotope and also the way the plant tissue is subjected to labeling through a chamber or culturing apparatus could create artifacts. This is especially important for MSI as the quality of data and images are critically dependent on the integrity of tissues imaged [37].

**Figure 2:**
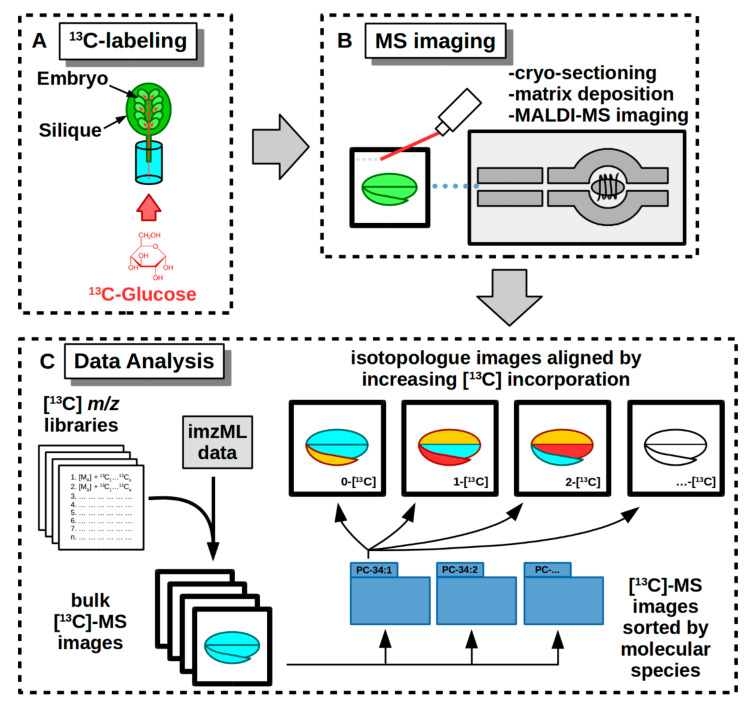
Workflow of a ^13^C-MSI experiment on developing embryos from *Camelina* and pennycress siliques. (**A**) Cut siliques are fed with media containing [U-^13^C]-glucose which is then metabolized and incorporated into the endogenous metabolites. (**B**) ^13^C-labeled seeds are dissected from siliques and prepared for MS imaging. (**C**) Raw data from MS imaging is converted to imzML format and is searched for images using [^13^C] *m/z* libraries. Isotopologue images are collected in bulk, sorted by molecular species, and then aligned by increasing ^13^C incorporation to show distribution of isotopically labeled molecular species.

To demonstrate ^13^C-MSI, we labeled *Camelina sativa* and pennycress embryos in siliques (developing fruit) with media containing [U-^13^C]-glucose (Figure 2A). Labeling in siliques requires that the [U-^13^C]-glucose is transported through the cut vasculature and then into the developing embryos, rather than into excised embryos directly, before the substrate is metabolized and the isotope incorporated within endogenous metabolites [8,9,10,11,12]. Previously, it was shown that metabolism within *Brassica* embryos is not significantly perturbed by labeling in siliques, which make it an ideal method for labeling plant embryos used for ^13^C-MSI [38]. Additionally, transport through the vasculature takes a longer time than uptake through the embryo directly which would therefore require a longer labeling time [38,39]. After some limited trials, we labeled for a period of several days to obtain sufficient enrichment for MS imaging.

Plant oilseed embryos synthesize and store lipids during development, are able to take in exogenous nutrients such as [U-^13^C]-glucose, and their metabolism is relatively stable for long periods of time, making them ideal candidates for ^13^C-MSI experiments [15]. Additionally, MALDI-MSI is particularly suited for imaging lipids in embryos, as has been shown in numerous other studies, because of the abundance of lipids and their heterogeneous distributions [22,26,28]. PC is an important intermediate in the synthesis of storage lipids and is also a favorable molecule for MSI analysis because of its inherent positively charged choline group. Other lipids, such as TG, that are neutral, may be suppressed and exhibit poor ionizability, which reduce their value in imaging with ^13^C-MSI. Ion distributions will be divided amongst the number of contributing adducts (e.g., TG is often found both as sodium and potassium adducts), and isotopologue peaks. One potential method to address ion suppression and poor ionizability is to adduct dope the matrix used to coat tissue sections used in ^13^C-MSI experiments to promote specific adduct formation and prevent multiple adducts of metabolites [40].

In this study, we imaged labeled molecular species of PC because it is an intermediate for TG synthesis within embryonic development, as well as an important membrane lipid, and it is well suited for MS imaging. Additionally, many other MS imaging studies have found different PC molecular species, varying in their fatty acid composition, are heterogeneously distributed among seed tissues, suggesting a difference in metabolism that may be revealed through isotopic labeling imaging [23,24,25,26,27,28]. Hence, imaging the molecular species of PC demonstrates the potential value of ^13^C-MSI for lipid metabolism studies.

### 2.3. Labeling and Harvesting Samples for MS Imaging

Labeling of embryos is done in a way that minimally perturbs the growth of the developing embryos so as to preserve as close to in vivo conditions as possible. Siliques are cut from the plant and then placed into sterile media containing the ^13^C-labeled substrate (Figure 2A). The media is made up of a buffer and a concentration of the labeled substrate that is similar to the substrate normally found from the mother plant. In this case we used 50 mM [U-^13^C]-glucose. Additionally, the labeling media can be supplemented with MS basal salts to support continued growth, especially for long labeling periods. The siliques are then left to incubate in labeling media under growth conditions that would be similar to what they would experience when grown *in planta*.

Two of the most important steps required when harvesting samples for ^13^C-MSI are: (a) halt all metabolic activity to capture the extent of labeling, and (b) maintain tissue integrity so that samples are suitable for MS imaging and differences in tissue-specific metabolism are kept separate.

Halting metabolic activity involves removing the siliques from media containing the [U-^13^C]-glucose, washing away the excess with buffer, and then fixing tissues within siliques using 4% p-formaldehyde in PIPES buffer (50 mM, pH 7.4) for several hours under vacuum, followed by several washes of PIPES buffer without formaldehyde. Flash freezing is not ideal for MSI analysis as it can damage tissues and result in poor MS image quality.

We reasoned that the delivery of labeled sugars to the developing embryos through the vascular system in the stem and silique represented a satisfactory simulation of natural seed development. We found that the seed age also contributed to optimal tissue integrity for sample processing. When labeled siliques were harvested earlier (e.g., approx. 15 day old *Camelina* embryos with less than 24 h of ^13^C-labeling), the seeds were underdeveloped, soft, and partially liquid endosperm, making the samples difficult to prepare and section for MS imaging. Similarly, pennycress embryos at 11 DAP are at the walking stick stage of development, consisting of mostly liquid endosperm and therefore also less suitable for MSI. *Camelina* and pennycress embryos at mid-development provided tissues with good structural integrity for MSI and active lipid metabolism for ^13^C-labeling. In the future, ^13^C-MSI studies with other systems will likely require trial and error to determine optimal MS imaging quality and sufficient isotopic incorporation. For general guidance in oilseed embryos (in siliques), we recommend introducing isotopically labeled substrates a couple of days after the walking stick stage, and to harvest after a period between three and five days for sufficient isotope incorporation into PC.

### 2.4. Cryo-Sectioning Biological Samples for ^13^C-MSI

Once samples have been harvested and fixed, they are then embedded in 10% gelatin to support the tissues and frozen at −80 °C. Prior to cryo-sectioning, the frozen, embedded tissues are equilibrated at −20 °C for 2–3 days.

**Figure 3:**
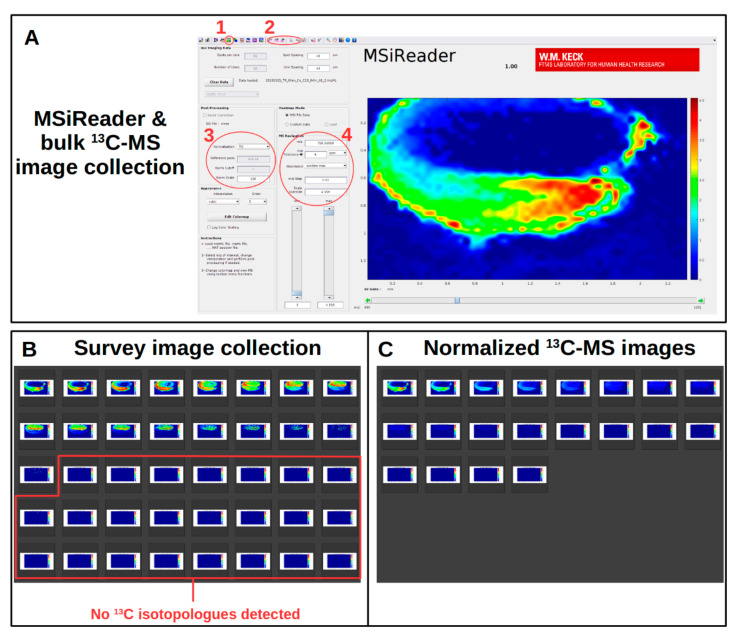
MSiReader software to analyze ^13^C-MSI data and photo gallery software to view collected images. (**A**) MSiReader is able to collect images using its “bulk processing” feature (1) and abundance values with “MSi Export” (2). Important settings include normalization (3) and the *m/z* and “m/z Tolerance” (4). The image depicted in the viewing window is [^13^C0] PC 34:2 from a *Camelina* embryo (embryonic axis on the bottom, red and green; cotyledons on the top, dark blue). (**B**) A survey image collection is first done to determine the extent of ^13^C-labeling and the isotopologue with the greatest abundance. (**C**) Normalized ^13^C-MS images are collected by discarding the *m/z* values without signal (as determined from the survey collection) and by locking the intensity value.

**Figure 4:**
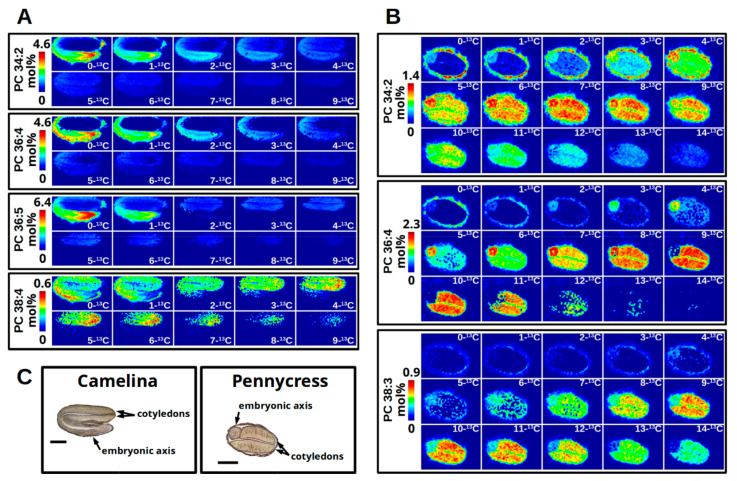
^13^C-MS imaging of PC from a developing *Camelina* embryo (**A**) and a developing pennycress embryo (**B**) isotopically labeled with [U-^13^C]-glucose for 64 h and 120 h, respectively. Brightfield images (**C**) of *Camelina* and pennycress embryo sections indicate anatomy and localization of cotyledon and embryonic axis tissues (scale bar = 500 μm). Isotopologues of ^13^C-MS imaging are aligned from least to most ^13^C incorporation from left to right and top to bottom. Each set of images for each molecular species is set to a colorimetric scale from dark blue (low abundance) to red (high abundance) representing mol% relative to the TIC of the imaging data. In *Camelina* (**A**), the greatest abundance for PC 34:2 (4.6), PC 36:4 (4.6), PC 36:5 (6.4) have low ^13^C incorporation and are localized to the embryonic axis (bottom). Isotopologues of greater ^13^C incorporation localize to the cotyledons (top) for PC 34:2 and PC 36:4, and exclusively for PC 36:5. PC 38:4 shows a low overall abundance for all isotopologues (0.6), but a more equal abundance between the isotopologues with greater ^13^C incorporation occurring in the cotyledons. In Pennycress (**B**), the isotopologue mol% abundance between the embryonic axis (left) and the cotyledons (right, most of embryo) for PC 34:2 and PC 36:4 are nearly equal, except PC 34:2 shows a similar isotopic enrichment between the two tissues whereas PC 36:4 shows different levels of enrichment (i.e., less in embryonic axis, more in cotyledons). PC 38:3 localizes only to the cotyledons with greater abundance at higher isotopologue values.

An important consideration for cryo-sectioning is the orientation of the embryo during sectioning. Since different tissues and possibly different parts of tissues may have altered metabolism at a given developmental stage, sectioning embryos in an orientation to capture both the cotyledonary and embryonic axis tissues in the same plane of section is preferable. *Camelina* embryo tissues are arranged such that cryo-sectioning longitudinally will show the embryonic axis adjacent to and contiguous with the two cotyledons, both outer and inner (as seen in Figure 3A and Figure 4C). In contrast, pennycress embryos are oriented with their cotyledons side by side with the embryonic axis in cross-section (as seen in Figure 4C), due to differences in seed anatomy and representative fields of view.

Once sections are taken, they are processed for MS imaging (Figure 2B). This involves lyophilization for 2–3 h or drying in a desiccator under vacuum overnight, followed by matrix application to sections of good quality (i.e., intact tissue, no obvious defects or tears in sections, correct orientation) as assessed under light microscopy. The tissue sections with deposited matrix are then ready for imaging.

### 2.5. MS Instrument Parameters

^13^C-MSI requires a high-resolution mass spectrometer capable of resolving masses resulting from the enrichment of ^13^C-isotopes. Multiple MS instruments have been used for MS imaging experiments, including linear trap quadrupole (LTQ), time of flight (TOF), Orbitrap, and Fourier transform-ion cyclotron resonance (FT-ICR) [4]. Fourier transform based MS instruments, such as Orbitrap and FT-ICR, are capable of increased mass accuracy by longer scanning times which increases the resolving power, the ability to separate two peaks of similar mass. A higher mass accuracy ensures the validity of *m/z* peak identification. Too low of a mass accuracy can result in misidentification of metabolites. Post-processing of collected data can also limit misidentification by setting a ppm (parts per million) mass accuracy threshold.

As the ^13^C-labeled substrate is incorporated into the endogenous metabolites the number of isotopologues increases (M + 1-[^13^C], M + 2-[^13^C]…M + n-[^13^C]). The *m/z* for many of these isotopologues will overlap, which is especially true for lipids as they occupy a narrow *m/z* window. Therefore, the resolving power of the mass analyzer used in a ^13^C-MSI experiment must be capable of separating *m/z* peaks of similar mass. The resolving power of various MS analyzers are approximately 40,000–60,000 for TOF, >100,000 for Orbitrap, and 1 × 10^6^ for FT-ICR [4]. Lipid molecular species may differ between each other by small masses, such as the addition or loss of a double bond resulting in a mass difference of 2.0157 amu. For ^13^C-labeled lipids, this difference in mass can become even smaller comparing ^13^C-labeled, unsaturated lipids with unlabeled, saturated lipids. For example, a monounsaturated lipid with two ^13^C-isotopes (2[^13^C] − 2[^12^C] = 2.0067 amu) differs from a saturated lipid with no ^13^C-isotopes (M + 2[H](saturation) = 2.0157 amu) by 0.0089 amu [41].

The smallest *m/z* difference between two peaks for each of the previously mentioned mass analyzers for a hypothetical mass at *m/z* 700 based on the given resolving powers would be between ±0.0117 and ±0.0175 amu for TOF, ±0.0070 amu for an Orbitrap, and ±0.0007 amu for FT-ICR. Since peaks differing by at least 0.009 amu would be required to differentiate ^13^C-labeled lipids of various saturation and unsaturation, high-resolution mass spectrometers like the Orbitrap or FT-ICR are required. This level of resolution would allow for accurate *m/z* peak identification. However, the *m/z* peaks of ^13^C-labeled metabolites of different classes can overlap to an even greater degree. For this reason, FT-ICR-MSI would provide the highest confidence of identification when using accurate mass alone. In this demonstration of ^13^C-MSI we use a MALDI-LTQ-Orbitrap from ThermoFisher set to 100,000 resolution with a stringent post-processing ppm threshold (described below) of the collected imaging data.

Other developments in instrumentation may lead to more confident *m/z* peak identification, such as the combination of MS/MS fragmentation imaging and ion mobility based separation [42].

In either case, careful attention should be taken to avoid misidentification when preparing MS instrument parameters, assuring it is calibrated appropriately and set to its highest resolving power prior to the MS imaging experiment. The difficulty in resolving the number of ^13^C-labeled peaks, especially when it comes to lipids, only further highlights the need for corroborating methods to confirm results from ^13^C-MSI experiments, such as side-by-side extract analysis by HPLC-MS/MS. Chromatographic separation by HPLC allows separating metabolites by polar/nonpolar characteristics, preventing *m/z* peak overlap, while MS/MS confirms metabolite identities and the extent of labeling, as shown in Supplementary Figure S1.

### 2.6. Data Analysis of ^13^C-Labeled MS Images

The MS imaging software used for ^13^C-labeled data requires the ability to view images of selected *m/z* values differing by ^13^C and ^12^C isotopes. Therefore, MSI software that searches data by non-isotopic molecular formula and its calculated accurate mass are unsuitable for ^13^C-MSI. ^13^C-labeling also dramatically increases the number of images to interrogate. For example, the molecular species PC 36:4 has a total of 44 carbons from the acyl chains, the glycerol backbone, and the choline headgroup. If each carbon were exchanged with a ^13^C-isotope, that would require 44 images for a single molecular species. Assuming an ideal ^13^C-MSI experiment were set up with five replicates of two different genotypes or conditions for a minimum of three time points, that would come to 1320 images for a single metabolite. Therefore, a procedure is needed for the analysis of ^13^C-MSI data that is manageable and capable of revealing biologically relevant information in a metabolic context. MSI software that is (A) capable of searching multiple *m/z* values and (B) produce multiple images in an automated manner are basic requirements for any MSI software used with isotopic labeling (Figure 2C).

The MSI software MSiReader, a Matlab-based software, has allowed the imaging of ^13^C-labeled sections [43]. The benefits of MSiReader over other MSI software for ^13^C-MSI are its accessibility and ability to output multiple images in an automated manner. MSiReader uses the open source imzML file format and is available across multiple operating systems (Windows, Mac, and Linux), which allows users of multiple different types of instruments from different manufactures to use the same data analysis methods. Bulk collection of images is also a benefit of MSiReader over other MSI software which either are incapable of automated bulk collection or require user input for each image collected. MSiReader collects images from multiple *m/z* values resulting in thousands of images of metabolites. There is no in-app way to view a collection of images at the scale required for isotopic labeling as done here, and options to customize image output are limited, thus requiring other software. However, a procedural workflow using MSiReader with additional software tools was developed here to accommodate ^13^C-labeled MS images and reveal metabolically informative imaging of tissue sections.

### 2.7. List of Software Used to Analyze ^13^C-Labeled MS Images

- MSiReader [43]
- Requires MATLAB Runtime or MATLAB
- Spreadsheet program (Microsoft Excel/Libreoffice/OpenOffice Calc)
- Photo gallery or image viewer browser (Microsoft Photos/Pix/gThumb)
- Text editor (Notepad/Gedit)
- ImageMagick [44]

### 2.8. Prior to Collecting or Analyzing MS Images

#### 2.8.1. Creating Libraries of Isotopologue *m*/*z* Values and Their Respective Adducts for Metabolite Classes of Interest

MSiReader produces an image from MS imaging data for a particular *m/z* value (Figure 3). In order to produce a number of isotopic images at once, batch images are collected from MSiReader using a list of calculated *m/z* values from a spreadsheet (Supplementary Table S1). For example, for PC the number of non-isotopically labeled metabolites would vary by the number of FA carbons and the degree of unsaturation. After calculating the initial monoisotopic *m/z* values for the non-isotopically labeled metabolites, the next step is to calculate the *m/z* value for every isotopologue possible by exchanging a ^12^C (12.000000 amu) for a ^13^C (13.003355 amu) within the calculated mass, a net increase of 1.003355 amu. For the PC lipid class, the total number of carbons to exchange will vary for each molecular species depending on the total number of C atoms in the acyl chains, since all headgroups should be the same for the class. An additional consideration is the mass of an adduct. Typically, PC ionizes as an H^+^ adduct, but can also ionize as K^+^ or Na^+^ adducts. Therefore, multiple libraries of PC isotopologue *m/z* values should be created for each adduct form possible; however, since isotopologues of different adducts are compared relatively, the adduct form with greater sensitivity should be used for imaging. Other adducts are able to confirm the labeling description and offer complementary support. Once these *m/z* libraries are calculated they are ready to use with MSiReader. The *m/z* values are searched either using the system clipboard or MSiReader’s library, and an image is collected for each value using the “batch processing” feature. The bulk of images are collected in a single directory which are then sorted using “mv” scripts, described in the next section.

#### 2.8.2. Custom Scripts for Moving Image Files to Appropriate Directories

Using the isotopologue *m/z* values described above, the bulk images are collected in a single directory. The next step is to sort the images into respective directories indicating the molecular species. Images collected through MSiReader’s “batch processing” feature saves each image as a PNG file with the filename of the *m/z* value. For example, an image collected for the H^+^ adduct of PC 34:2 that is unlabeled would have an *m/z* of 758.56998. The resulting image filename would be: 758_56998.png. To sort through the bulk images, a series of terminal commands targets and moves the images of specific *m/z* values corresponding to the isotopologue for each molecular species to a directory named after that same molecular species. For example, the Linux command “mv” will move each of the 43 images of PC 34:2 to a target directory with the name of the molecular species (“PC-34-2”). Combining these individual commands for each molecular species into a single script is the basis for sorting through the bulk of images to be collected (Supplementary File S1).

### 2.9. Initial Survey for Metabolites, Adduct Forms, and Extent of ^13^C-Labeling

#### Viewing ^13^C-Labeled MS Images within MSiReader

Before collecting all of the images from each replicate and time point involved in the ^13^C-MSI experiment, an initial survey is done to determine the level of ^13^C-labeling, the adducts formed by the metabolites of interest, and the molecular species that were detected within each metabolite class. This initial survey reduces the need to collect images of molecular species or isotopologues that lack signal, saving time and computer memory. Loading MS imaging data in the imzML format into MSiReader allows the researcher to search for metabolites via their calculated accurate *m/z* (Figure 3A, circle 4), referring back to the libraries of *m/z* values discussed above [43,45]. Settings for normalization and abundance calculations in the “Post Processing” and “MS Navigation” panels of the MSiReader program will depend on the nature of the data collected, determined by the instrument used to collect the imaging data. For our purposes, imaging is normalized to the TIC of each pixel (Figure 3A, circle 3) and set to a value of “100”, and “Abundance” is set to the maximum of each peak.

An important parameter to set here is the “*m*/*z* Tolerance” (Figure 3A, circle 4), which will specify the mass accuracy required for imaging to appear. As noted in the discussion above, an *m/z* difference of 0.009 amu separates a lipid lacking a double bond versus another that has an additional double bond and two ^13^C isotopes. These small mass differences, between varying levels of unsaturation and the number of ^13^C isotopes incorporated, are common among lipids. As an example, in order to differentiate between PC 34:2 (*m/z* 758.56998) and [^13^C2] PC 34:3 (*m/z* 758.56104), the “*m*/*z* Tolerance” would have to be lower than 11.8 ppm. If the “*m*/*z* Tolerance” is set to 5 ppm with mass accuracy potential peak overlaps can be avoided.

With the desired “Post Processing”, smoothing, and other parameters set, images are then collected for all isotopologues of a molecular species including potential adduct forms. Images are collected via MSiReader’s batch processing feature (Figure 3A, circle 1) which saves an image for each *m/z* value in the calculated *m/z* library (described above). Once the images are collected, they are sorted by the scripts into molecular species directories.

The sorted images are then assessed using a photo gallery program that is able to align the collected images alphanumerically. An example is shown in Figure 3B. In this initial survey, the type of adducts, the different molecular species, and the extent of ^13^C-labeling can all be determined. Knowing this information, a revised and, therefore, reduced number of *m/z* values can be assembled to collect the remaining images for other replicates and time points. One important note is that the images collected in this initial survey leave the scale of the color intensity to be optimized by the MSiReader program based on the signal intensity of each individual image. However, to give an accurate representation of the relative abundance for each isotopologue, the scale should be locked to the maximum value as determined by the most abundant isotopologue. This will ensure the intensity scales are all the same between isotopologues and provide a direct relative comparison of the images collected (Figure 3C, described below).

### 2.10. Collection of Normalized ^13^C-Labeled MS Images

#### 2.10.1. Revising *m*/*z* Library Values for Other Replicates

The initial survey will reveal the molecular species that are present and the extent to which they are isotopically labeled. To collect the remaining images from the other replicates, time points, and genotypes or conditions, a modified library of *m/z* values is created to reflect only those isotopologues and molecular species present. This new modified and shortened library of *m/z* values is used to collect the new images.

Normalized ^13^C-MS images are collected for each molecular species separately using the modified library and by setting the intensity of the color scale (Figure 3A, circle 4, labeled as “Scale Override”) to the highest value found in the initial survey. Images collected in this way show which isotopologue is most labeled and how others gradually diminish in intensity with less ^13^C-labeling. Collecting images in this way normalizes to a shared intensity scale within the molecular species.

An alternative approach is to collect images while allowing MSiReader to optimize the intensity for each isotopologue (similar to that shown for the survey image collection in Figure 3B). Images collected in this way are helpful for showing localization of the different molecular species and their isotopologues.

#### 2.10.2. Organizing Collected Images for High-Quality Figures and Interpreting Labeling Patterns

With a number of images collected, a high throughput method to assemble the images into a grid-like view with the number of incorporated ^13^C-isotopes increasing from left to right and top to bottom is to make use of the open source software ImageMagick [44]. The “chop” and “montage” features of ImageMagick allow aligning the isotopologue images into a grid as a new combined PNG image for each molecular species of interest. Figure 4 shows the result of montaging the isotopologue images for selected PC molecular species from 64 h ^13^C-labeled *Camelina* embryos (Figure 4A) and 120 h ^13^C-labeled pennycress embryos (Figure 4B). The ^13^C-MS imaging of *Camelina* embryos (Figure 4A) shows that both PC 34:2 and PC 36:4 have a similar abundance (a max intensity scale set to 4.6), a similar level of ^13^C enrichment with isotopologues up to 4-[^13^C] before tapering off, and have similar localizations. In both molecular species there is a greater abundance of less ^13^C-labeled isotopologues in the embryonic axis, and virtually none of PC 34:2 from 0-[^13^C] to 2-[^13^C] and none of PC 36:4 from 0-[^13^C] to 4-[^13^C] in the cotyledons. However, cotyledons show an abundance of the more ^13^C-labeled isotopologues of those same molecular species. In contrast, there appears to be no ^13^C-labeled PC 36:5 in the embryonic axis, with the intensity at 1-[^13^C] accounted for by the natural abundance of ^13^C, and is instead only found in the cotyledons. The isotopologues of the higher molecular weight PC 38:4 appear to be principally in the cotyledons with similar abundances until approximately 8-[^13^C] but at a much lower total abundance in comparison to the other PC molecular species (an intensity scale of 0.6 vs. 4.6 or 6.4).

The enhanced PC 38 labeling in cotyledons is consistent with a more enriched acetyl-CoA pool in the cytoplasm that is the substrate for elongation to produce 20 or longer carbon acyl chains that would be required for this PC molecular species. On PC 38 the elongated acyl chains would be paired with either a 16 or 18 carbon acyl chain and could be shuttled rapidly through PC for storage oil biosynthesis in cotyledons relative to the embryonic axis. The lack of embryonic axis labeling for the same lipid may suggest that this organ is producing less of this species and thus not labeled within the duration of the experiment. These results were partially confirmed through independent experiments with *Camelina* analyzed by LC-MS/MS where the embryonic axes tissues were separated from the cotyledons prior to isotopologue quantification. Inspection of the MS2 data for paired PC 38:4 fatty acids indicated two combinations of acyl chains including 18:3_20:1 and 18:2_20:2. The FA 20:2 does not represent a significant storage oil acyl chain in *Camelina* and some labeling in the embryonic axis may indicate biology specific to that tissue that is a small fraction of total seed. The relative labeling in 18 carbon fatty acids was higher in the cotyledon than in the embryonic axis as was labeling in FA 20:1, a significant acyl chain in *Camelina* (Table 1). The results are consistent with enhanced production of these acyl chains that transit through PC prior to inclusion in storage lipid.

The ^13^C-MS imaging of pennycress embryos (Figure 4B) shows greater overall ^13^C-label incorporation with a longer labeling time (120 h) compared to the *Camelina* embryos (64 h) (Figure 4A). Similar to the *Camelina* embryos, the PC 34:2 and PC 36:4 found in pennycress localize to both the embryonic axis and cotyledons, but there is greater ^13^C enrichment in the cotyledons than the embryonic axis for PC 36:4 (i.e., less ^13^C-label incorporation in the embryonic axis compared to cotyledons). For either molecular species the greatest amount of ^13^C-label incorporated appears to reach a maximum of about [^13^C12] PC 36:4 and [^13^C14] PC 34:2. The isotopologue with the greatest abundance for PC 34:2 for pennycress appears to be about 7-[^13^C] for both tissues whereas for PC 36:4 it is approximately 6 or 7-[^13^C] in the embryonic axis and approximately 9 or 10-[^13^C] in the cotyledons. Differences in the abundance of isotopologues in different tissues may reflect a greater accumulation for the same molecular species in the cotyledons compared to the embryonic axis. Additionally, similar to the PC 38:4 in *Camelina*, the PC 38:3 in pennycress appears to localize primarily to the cotyledons and show greater ^13^C-label incorporation than the lower weight PC molecular species but at an overall lower total abundance (max intensity scale set to 0.9 vs. 1.4 for PC 34:2 or 2.3 for PC 36:4).

Comparing images from control embryos either before ^13^C-labeling (0 h time point) or incubated in media with unlabeled substrates are important controls to show the lack of isotopologue images (see Supplementary Figure S2).

### 2.11. Interrogating Tissue Specific ^13^C-Labeling Patterns

**Figure 5:**
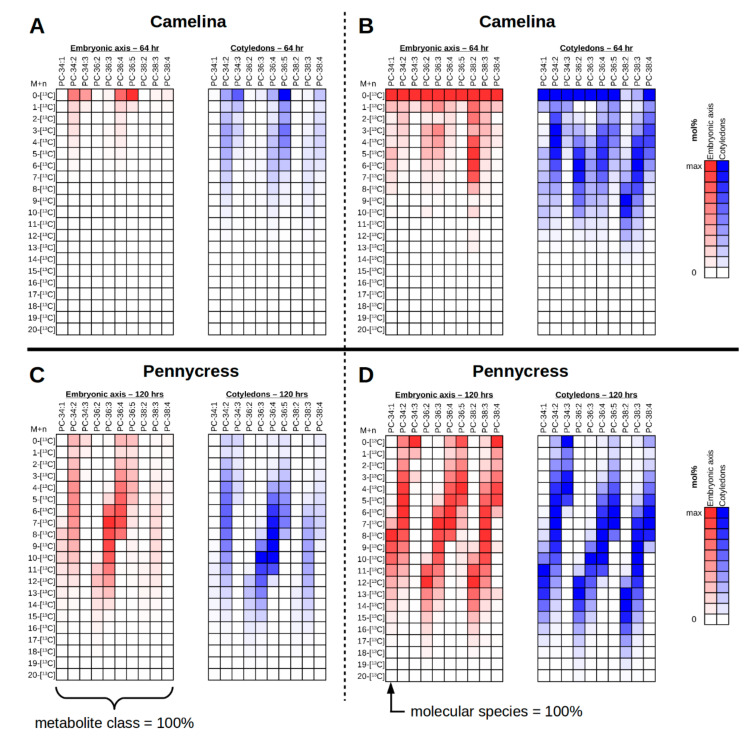
Isotopologue mol% abundance for PC of the different tissues of a ^13^C-labeled *Camelina* embryo (**A**,**B**) and a ^13^C-labeled pennycress embryo (**C**,**D**). Mol% abundance values are exported from MSiReader using “MSi Export” and ROI selection of either the embryonic axis (red) or the cotyledons (blue). Summed values are normalized either to the entire metabolite class (**A**,**C**) to depict relative abundance, or for each individual molecular species (**B**,**D**) to depict ^13^C-labeling patterns. The natural ^13^C abundance was corrected using IsoCorrectoR [46].

To interrogate the isotopologue mol% abundances for a particular molecular species and metabolite class, the polygon ROI (region of interest) tool from MSiReader (Figure 3A, circle 2) can be used to select a particular tissue region, e.g., cotyledons or embryonic axis in the imaged plant embryos, followed by “MSi Export” to export the abundance values for each of the pixels selected within the ROI. The *m/z* values used to export are for each isotopologue of each molecular species desired to relatively quantify. The output is the abundance for each *m/z* value for each pixel within the ROI, which include all of the isotopologues of every molecular species of interest collected at once. Values for each molecular species can then be summed and then normalized to the total abundance across all of the isotopologues for all of the molecular species as shown in Figure 5A (*Camelina*) & 5C (pennycress). This depiction is useful to show the relative abundance of molecular species and their respective isotopologues. For example, the most abundant PC molecular species in ^13^C-labeled *Camelina* embryos are PC 34:2, PC 34:3, PC 36:4, PC 36:5, and PC 36:6 (Figure 5A). However, when the embryonic axis (red) and the cotyledonary (blue) tissues are compared, there is a greater abundance of ^13^C-labeled isotopologues of PC 34:2, PC 36:4, and PC 36:5 in the cotyledons than the embryonic axis. In the ^13^C-labeled pennycress embryos, the most abundant PC molecular species are PC 34:2, PC 36:3, and PC 36:4, with additional contribution from PC 34:1, PC 36:2, PC 36:5, and PC 38:3 (Figure 5C). Here, however, the PC has incorporated much more ^13^C-label and are higher molecular weight isotopologues. Like the *Camelina* embryo, the cotyledonary tissues (blue) show a slight increase in ^13^C-label incorporation compared to the embryonic axis (red). Together these complementary pieces of evidence would suggest that the ^13^C-labeled substrate used to label these embryos was preferentially incorporated in PC molecular species in the cotyledons compared to the embryonic axis, which may indicate greater flux compared to the embryonic axis.

The abundance values also can be normalized within each individual molecular species rather than the entire metabolite class (Figure 5B, *Camelina,* & Figure 5D, pennycress). This will give the relative mol% distribution of the isotopologues for a particular molecular species and show which is of the greatest abundance. This type of representation is especially useful for determining labeling patterns within molecular species relative to the metabolite class. In the ^13^C-labeled *Camelina* embryos, there appears to be an overall greater amount of ^13^C enrichment in cotyledonary tissues (blue) relative to the embryonic axis (red), with the highest ^13^C isotopologue reaching 12-[^13^C] (Figure 5B). In the ^13^C-labeled pennycress embryos there is only a slight increased amount of ^13^C enrichment in the cotyledonary tissues compared to the embryonic axis, reaching a maximum ^13^C isotopologue of approximately 18-[^13^C]. Additionally, the pattern of ^13^C-labeling suggests that there is greater ^13^C enrichment in more saturated PC species (e.g., PC 34:1, PC 36:2, PC 36:3, PC 38:2, PC 38:3) compared to more unsaturated PC species (e.g., PC 34:3, PC 36:5, PC 38:4) in both plant embryos, which is consistent with MS2 data of PC 38:4 in the cotyledons of *Camelina* described in Table 1 where FA 18:2 is more labeled than FA 18:3, and FA 20:1 more than FA 20:2. Thus, this may reflect that desaturation is a relatively slower process than incorporation of the acyl chains. There also appears to be a trend of greater ^13^C enrichment with larger molecular weight PC molecular species (i.e., those with elongated, very long chain FAs), as seen by comparing [^13^C4] PC 34:2, [^13^C6] PC 36:4, and [^13^C10] PC 38:2 or [^13^C7] PC 38:3 in the cotyledons of *Camelina* (Figure 5B, blue), and comparing [^13^C12] PC 34:1, [^13^C14] PC 36:2, and [^13^C15] PC 38:2 in the cotyledons of pennycress (Figure 5D, blue). This could indicate that acetyl groups are more readily incorporated by elongation at the ER than by de novo fatty acid synthesis in the plastid. Alternatively, very long chain fatty acids may have an altered “resident time” in PC than shorter chain acyl groups. In any case, plots such as these help to visualize labeling patterns that might otherwise go unnoticed in the ^13^C-MS images, and will help develop testable hypotheses that can be addressed through further temporal analyses with additional metabolites.

We investigated the incorporation of ^13^C-labeled carbon in the last acetyl fragment of palmitic acid (FA 16:0) and erucic acid (FA 22:1) to understand the labeling patterns between de novo fatty acid synthesis in plastids and fatty acid elongation in the cytosol, respectively. Consistent with MSI imaging data, we found greater ^13^C enrichment in cotyledons than in the embryonic axis (Supplementary Figure S3). However, there was no significant difference in the incorporation of ^13^C between FA 16:0 and FA 22:1 in the cotyledons, and only a slightly significant increase of ^13^C enrichment in FA 22:1 of the embryonic axis relative to FA 16:0. This suggests that differences in ^13^C-labeling between de novo fatty acid synthesis and fatty acid elongation are not nearly as obvious as that found with unsaturation. Instead, it may again suggest that “resident time” of acyl groups in PC may be a part of the explanation for different labeling patterns for long chain fatty acids or polyunsaturated fatty acids in PC. Further, it appears that there is a tissue-specific difference in the appearance of very long chain isotopologues, especially in *Camelina*, where 20 carbon fatty acid containing PC species are less labeled in the embryonic axis relative to the cotyledons (Figure 5A). These tissue differences in labeling of 20 carbon fatty acid containing PC are evident in pennycress as well (Figure 5C), and explain the resulting heterogeneity noted in unlabeled *Camelina* and pennycress embryos (Supplementary Figure S2).

## 3. Materials and Methods

### 3.1. Chemicals

Uniformly [U-^13^C]-glucose was purchased from Isotec (now available from Sigma, Milwaukee, WI, USA). Murashige and Skoog (MS) basal salts and Gibberellins (A4 + A7) were purchased from PhytoTechnology Laboratories. Porcine gelatin, p-formaldehyde, PIPES, HEPES, N-butylamine, and 2,5-dihydroxybenzoic acid were obtained from Sigma. Hexanes and isopropanol were purchased from ThermoFisher Scientific (Fair Lawn, NJ, USA).

### 3.2. Plant Material

Growth conditions for *Camelina sativa* plants were temperature of 22/20 °C (day/night), 40–50% relative humidity, and 14 h day/10 h night photoperiod. Day light condition was maintained at minimum 200 µmol m^−2^ s^−1^ coming from a combination of 600 W high pressure sodium and 400 W metal halide bulbs, while the light intensity was varied up to 400 µmol m^−2^ s^−1^ in bright sunlight. Fifteen days old siliques were harvested for culturing in ^13^C-isotope containing media.

Seeds of pennycress MN106 accession were obtained from Dr. David Marks, University of Minnesota. Seed germination, plant growth, and daily flower tagging were performed as previously described [47].

### 3.3. ^13^C-Labeling and Culturing Conditions

Cut siliques from *Camelina* were placed in 5 mM MES media containing [U-^13^C]-glucose for 64 h under continuous light. Following ^13^C-labeling, siliques were then fixed by vacuum infiltrating in 4% p-formaldehyde in 50 mM PIPES, pH 7.2 buffer for 2 h followed by 3X washes of PIPES buffer without p-formaldehyde for 15 min each. Fixed siliques were stored in the last wash and placed at 4 °C until prepared for embedding.

For labeling of pennycress, 16 DAP MN106 siliques were collected from the greenhouse 5–6 h into the day cycle and carried on ice to a clean bench. Siliques were sterilized with 5% bleach for 2–3 min and washed 4–5 times with sterile water. The pedicel was recut in water and wiped with an autoclaved Kimwipe before incubating in 0.71 mL of unlabeled media (unlabeled 50 mM glucose, 50 mM HEPES buffer and 3 mg/mL MS basal salt) for 24 h before transferring to ^13^C-labeling media in which unlabeled glucose was replaced with 100% [U-^13^C]-glucose. To insert a single silique pedicel into the culture solution, a small hole was perforated into the cap of sterilized PCR tubes (Fisher Scientific). PCR tubes were transferred to a 200 μL tips box containing two holes for air circulation. Wet sterile filter paper was placed at the bottom of each box, and holes were taped with Millipore tape for continuous circulation of air. Culture tubes inside the box were incubated for 120 h in a Percival chamber under 16 h light and 8 h dark at 22 °C. The light intensity was set to 200 μmole m^−2^ s^−1^. At the end of incubation, siliques used for ^13^C-MSI were handled similarly as the *Camelina* siliques described above. For samples used to determine the labeling abundance of the C1-C2 acetyl fragment, 15 embryos were quickly isolated from siliques, separated into axis and cotyledons under stereo microscope and collected into pre-chilled 2 mL tubes. Tubes were frozen in liquid nitrogen to quench metabolism, and lyophilized for approximately three days. After lyophilization, the dry weight of tissues was recorded and samples stored at −80 °C until further analysis.

### 3.4. Fatty Acid Extraction and Derivatization with N-Butylamine from Pennycress Embryos

Fatty acids were extracted and derivatized with N-butylamine [48]. Dried fatty acid butyl amide (FABA) derivatives were resuspended into 0.25 mL hexanes and analyzed using GC-MS as previously described, corresponding to butyl amide derivatives of palmitic and erucic acids [8]. GC-MS data were acquired and processed using Xcalibur software.

### 3.5. MS Imaging

#### 3.5.1. Embedding and Cryo-Sectioning Embryos

Embryos were dissected from fixed siliques and then embedded in 10% gelatin held at 40 °C. Once the gelatin had solidified, excess gelatin was trimmed around the embryo prior to freezing at −80 °C for at least 24 h before transferring to −20 °C for 2 to 3 days prior to cryo-sectioning.

Longitudinal sections of embryos were taken at 30 μm thickness using a CM1950 cryomicrotome (Leica Biosystems, Buffalo Grove, IL, USA) and then thaw mounted on to Superfrost Plus microscope slides (Fisherbrand ca. no. 12-550-15, Waltham, MA, USA). Collected sections were immediately lyophilized for 2 to 3 h and then held in a benchtop desiccator under vacuum until ready for MS imaging.

#### 3.5.2. Matrix Application and MALDI-MS Imaging Instrument Parameters

Sections were inspected under light microscopy to determine quality (i.e., intact tissue, no cosmetic defects) and coated with 2,5-dihydroxybenzoic acid (DHB) by sublimation as described by Hankin et al. [49].

Matrix-coated sections were imaged using a MALDI-LTQ-Orbitrap-XL mass spectrometer (ThermoFisher Scientific, San Jose, CA, USA). All sections to image PC were collected in positive ionization mode. The MALDI ionization parameters were set as: 12 μJ/pulse, 10 laser shots per step, and 40 μm step size. The Orbitrap mass analyzer was set to collect from *m/z* 600 to 1200 with a set resolution of 100,000. LTQ Tune Plus and Xcalibur software (Thermo Scientific) were used to operate the instrument and collect the raw data.

#### 3.5.3. Extraction, Chromatographic Separation and Instrumentation Used for LC-MS/MS Analysis

Unlabeled and 64 h labeled *Camelina* tissue separated into embryo axis and cotyledon, were extracted using a phase separation method, previously described with slight modifications [50]. Briefly, 700 µL of 3:1 (*v*/*v*), chloroform: methanol solution was added to dried lyophilized tissue along with 10 µL of EquiSPLASH^®^ (Avanti polar lipids, Birmingham, AL, USA) as labeled internal standard mix. Samples were incubated at 4 °C for two hours and centrifuged at 14,000 rpm for 10 min immediately after addition of 300 µL of ddH_2_O to achieve phase separation. The upper aqueous layer was carefully removed and 200 µL of methanol was added to the remaining organic phase containing lipids. Samples were centrifuged to pellet debris and the organic phase was collected, and then dried before re-suspending in 200 µL of solvent B used in LC-MS/MS analysis.

The chromatographic conditions used for the separation of PC molecular species were similar to those described by Hummel et al., 2011 [51]. Two microliters of sample was injected on a C8 reversed phase column (100 mm × 0.5 mm × 1.7 µm particles, Higgins Analytical Inc., Mountain View, CA, USA), using the loading pump of the Dionex UltiMate™ 3000 RSLC nano system. The chromatographic gradient using 1% 1M NH_4_Ac, 0.1% acetic acid in ddH_2_O as solvent A and 1% 1M NH_4_Ac, 0.1% acetic acid in 7:3 (*v*/*v*) acetonitrile: isopropanol as solvent B, were as follows; 0–1 min 55% B, 4 min 75% B, 12 min 89% B, 15 min 99% B, 18 min 99% B, 18.5 min 55% B and 30 min 55% B. A flow rate of 40 µL per min was maintained throughout the run. Mass spectra at both MS^1^ (450–1200 *m/z*) and MS^2^ (50 precursor ion *m/z*) levels were collected using an Orbitrap Fusion Lumos Tribrid mass spectrometer with the resolution of MS1 scans set to 240,000 and that of MS2 scans set to 140,000. Ions were collected in negative mode with maximum injection time and the target ion count restricted to 200 ms and 1.0 E6, respectively. MS2 data were acquired in a data dependent manner with collision achieved in HCD cell at a normalized energy of 35 eV. Fatty acid fragment ion intensities of all isotopologues of desired PCs were manually integrated and data analyzed in Microsoft Excel (Supplementary Table S2). IsoCorrectoR was used for natural abundance correction [46].

### 3.6. Data Analysis

Raw data was exported from ImageQuest software (ThermoFisher Scientific) into the open source format imzML [45]. Data in the imzML format were then loaded into MSiReader (version 1.00) with the following settings: Normalization to TIC, Normalized Scale set to 100, smoothing set to 5, and an *m/z* Tolerance of 5 ppm. Accurate *m/z* values for each molecular species of PC and potential ^13^C isotopologue as H^+^ adducts were previously calculated before collecting images of each using the batch processing feature of MSiReader. Isotopologue images for each PC molecular species were then sorted into their respective directory labeled with the PC molecular species name using “mv” scripts. For the initial survey of images, the sorted images were then viewed using an image gallery program to determine extent of ^13^C-labeling and the mol% abundance of the isotopologue images. In the collection of normalized images, the isotopologue with the greatest mol% value for each PC molecular species in the survey collection was used to set the scale of collected images in MSiReader. Normalized images were then cropped and montaged using ImageMagick software [44]. All ^13^C-MSI data analysis was conducted on a computer running Linux Mint 18.2 64-bit (kernel: 4.8.0-53, processor: Intel Core i5-3570k 3.40 GHz × 4, ram: 8Gb).

To depict the isotopologue mol% as colorimetric plots, abundance values were exported from MSiReader using the same calculated *m/z* values described above for each isotopologue of each molecular species. The ROI tool was used to select either the embryonic axis or cotyledonary tissues. Abundance values from each pixel were summed together for each isotopologue. Summed values were then corrected for the natural ^13^C abundance using the R-based tool IsoCorrectoR with R (version 3.6.3) and RStudio (version 1.1.423) [46]. The results were then colored (red for embryonic axis, blue for cotyledons) from low (white) to high (full color), normalized either to the summed abundance values for the entire PC lipid class, giving relative abundance (Figure 5A), or to the summed abundance values of the particular molecular species, giving the relative abundance of each isotopologue within a molecular species and the overall ^13^C-labeling patterns (Figure 5B).

## 4. Conclusions

Here we demonstrate a method for conducting and analyzing ^13^C-MSI experiments by isotopically labeling plant embryos in siliques. A workflow is described to process the resulting data to visualize the isotopically labeled metabolites and show their relative abundance using the MSiReader software. [U-^13^C]-glucose was used to label *Camelina sativa* and pennycress embryos for 64 and 120 h, respectively, to incorporate sufficient labeling into molecular species of the membrane lipid and TG intermediate―PC. Following the labeling period, embryos were processed in the same manner as unlabeled tissues for MS imaging. The resulting ^13^C-MSI data was large and complex, requiring procedures to filter through the potential isotopologues of metabolites of interest to produce biologically meaningful and interpretable results. In embryos of both plants, imaging and comparisons of the various isotopologues for ^13^C-labeled PC showed a heterogeneous distribution of isotopologues with greater ^13^C incorporation localized to the cotyledons compared to the embryonic axis, likely consistent with greater flux of carbon to the cotyledons at this developmental stage. Plotting the isotopologue mol% values colorimetrically revealed the additional information provided through ^13^C-labeling patterns. The labeling patterns in both systems show a trend toward greater ^13^C incorporation in more saturated PC molecular species and higher molecular weight PC molecular species than unsaturated or shorter chain FAs containing PC, which may suggest label was used for elongation but had not been used extensively in all desaturation reactions. Alternatively, this pattern may reflect differences in “residence time” for molecular species in the PC pool based on their acyl composition. Resolving these possibilities will require expansion of these procedures to include additional time points and additional lipid metabolites upstream and downstream PC acylation.

^13^C-labeling is likely being directed through glycolysis to produce labeled glycerol (3C) and acetyl-CoA (2C), which enters both the de novo FA synthesis in the plastid and the FA elongation pathway in the cytosol (see Figure 1). Higher molecular weight PC species, which would have elongated FAs, show greater ^13^C-labeling than the lower molecular weight PC species, as seen from the ^13^C-MSI data (Figure 5). This slightly higher ^13^C-labeling in higher molecular weight PC might be a reflection of the ^13^C entering the FA elongation pathway. However, analysis of the C1-C2 acetyl fragments from fatty acid butyl amides of FA 16:0 and FA 22:1 suggests that elongation is not significantly higher than de novo FA synthesis (Supplementary Figure S3). Additionally, given that the more saturated molecular species show greater ^13^C-labeling than the more unsaturated molecular species, desaturation of FAs on PC may indicate a slower or longer process and would therefore have less opportunity to incorporate ^13^C-isotope either by FA elongation or by re-incorporation into glycerolipids (with labeled glycerol and/or labeled choline) following acyl editing (e.g., LPCAT, PDAT, PDCT, and other lipases, Figure 1). While such conclusions require further evidence, including analysis of mutants in PC and TAG biosynthesis, HPLC-MS/MS of extracts from dissected embryonic tissues and MS/MS analysis of labeled metabolites to determine what parts of PC have been labeled, here we demonstrate that combining isotopic labeling of lipids and MSI is able to provide new information about tissue-specific differences in metabolism that would otherwise remain hidden in typical extraction-based methods. Such examination of these metabolic differences will help to provide explanations for the heterogeneity of metabolites observed within different tissues, and will continue to uncover the complex spatial dimension to metabolism [22,23,24,25,26,27,28].

The combination of isotopic labeling and MS imaging is still in its infancy, but as technology and experimental methods begin to develop and mature, the result will be a collection of complex data sets requiring a workflow for processing the data into meaningful results. This procedure for processing ^13^C-MS imaging data is one such method that could be used with other stable isotopes or other isotopically labeled samples using currently available tools. Future fluxomics MS imaging studies will add a novel view on metabolomics that will show a truer picture of the spatial heterogeneity of metabolism within tissues and their individual rates of reactions than either method could reveal on its own.

## Figures and Tables

**Table 1:** Average labeling in fatty acids within PC 38:4 from *Camelina.*

64 h Labeling * in PC 38:4		
Paired Fatty Acids	Cotyledon (%)	Embryo Axis (%)
FA 18:2	10.37	6.54
FA 20:2	2.76	5.39
FA 18:3	7.11	1.28
FA 20:1	8.08	3.77

* calculation for labeling within fatty acids is presented in Supplementary Table S2, values represented in percentage.

## Data Availability

Data sharing is not applicable to this article.

## Supplementary Materials

The following are available online at https://www.mdpi.com/2218-1989/11/3/148/s1, Figure S1: HPLC-MS/MS of PC 36:5 from Camelina, Figure S2: MSI of 0 h labeled and unlabeled Camelina and pennycress embryos, Figure S3: Labeling abundance (%) per carbon of C1-C2 acetyl fragment in pennycress embryos, Table S1: m/z library for H+ adducts of PC molecular species, Table S2: Calculations for isotopologue distribution and average labeling of fatty acid pairs in PC 38:4 using MS2, File S1: mv script for PC isotopologue images.
