# Supplementary material for: Analyzing Mass Spectrometry Imaging Data of 13C-Labeled Phospholipids in Camelina sativa and Thlaspi arvense (Pennycress) Embryos
Source: Metabolites. 2021 Mar 4;11(3):148. doi: 10.3390/metabo11030148 (PMC7999836; doi:10.3390/metabo11030148)
Supplement: Supplementary file 1 [file metabolites-11-00148-s001.zip › metabolites-1128787-SP-revision/supplementary_materials/13C-MSI_Methods_Metabolites_supplementary_figures.pdf]

metabolites

Supplementary Figures

Analyzing Mass Spectrometry Imaging Data of ^{13}C-labeled Phospholipids in *Camelina sativa* and *Thlaspi arvense* (Pennycress) Embryos

Trevor B. Romsdahl [1], Shrikaar Kambhampati [2], Somnath Koley [2], Umesh P. Yadav [1], Ana Paula Alonso [1], Doug K. Allen [2,3,*] and Kent D. Chapman [1,*]

[1] Department of Biological Sciences & BioDiscovery Institute, University of North Texas, Denton, TX 76203, USA; trevor.romsdahl@unt.edu (T.B.R.); umeshprasad.yadav@unt.edu (U.P.Y.); Anapaula.Alonso@unt.edu (A.P.A.)
[2] Donald Danforth Plant Science Center, St. Louis, MO 63132, USA; SKambhampati@danforthcenter.org (S.K.); SKoley@danforthcenter.org (S.K.)
[3] United States Department of Agriculture, Agriculture Research Service, St. Louis, MO 63132, USA
* Correspondence: doug.allen@usda.gov (D.K.A.); Kent.Chapman@unt.edu or chapman@unt.edu (K.D.C.); Tel.: +1-940-565-2969 (K.D.C.)

Supplementary Figures

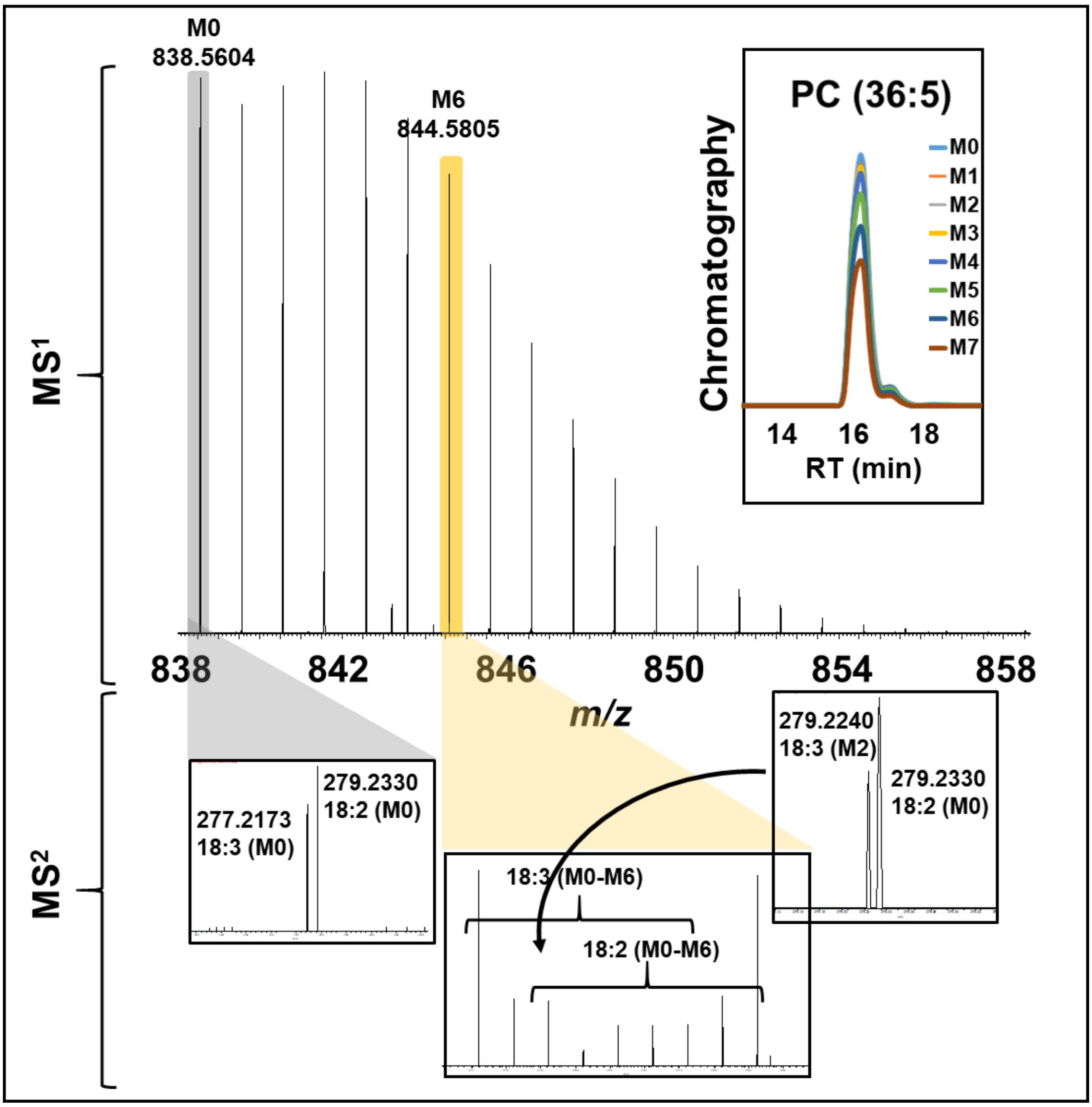

Supplementary Figure S1. HPLC-MS/MS of PC 36:5 from *Camelina*. PC 36:5 that was chromatographically separated, from other lipid species with several isotopologues that co-elute with the same retention time (RT). High resolution MS resolves the different isotopologues as shown highlighted in gray, M0 (838.5604), and yellow, M6 (844.5805), in the MS^1 scan. MS^2 fragmentation reveals unlabeled 18:3 and 18:2 FAs in M0, while different isotopologues of 18:3 and 18:2 FAs are found in the M6 peak of PC 36:5. High resolution at MS^2 level is also able to resolve ^{13}C-labeled 18:3 at M2 (279.2240) relative to unlabeled (M0) 18:2 (279.2330).

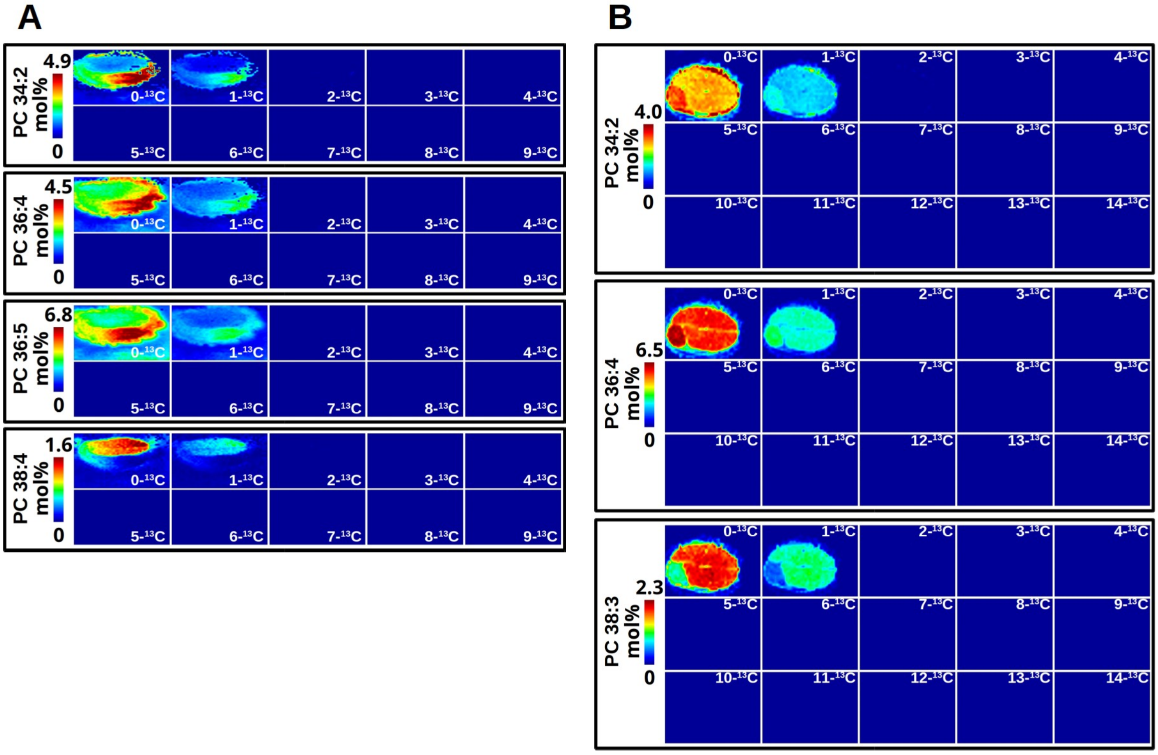

Supplementary Figure S2. MSI of PC from a developing *Camelina* embryo (**A**) at 0 h of ^{13}C-labeling and a developing pennycress embryo (**B**) fed for 120 h in media without a ^{13}C-labeled substrate. Both a 0 h time point (**A**) and an unlabeled control (**B**) are appropriate controls for a ^{13}C-MSI experiment, and both show a lack of ^{13}C incorporation for the imaged PC molecular species. The signal at 1-[^{13}C] is due to the natural abundance of ^{13}C.

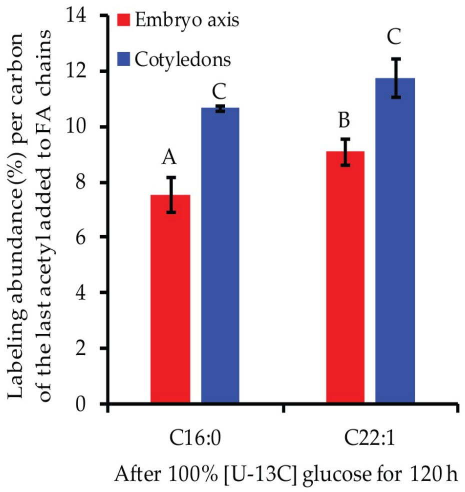

Supplementary Figure S3. Labeling abundance (%) per carbon of C1-C2 acetyl fragment in pennycress embryos from siliques fed with 100% uniformly ^{13}C-labeled glucose for 120 h. For the labeling information of plastidic and cytosolic acetyl-CoA, McLafferty fragments of palmitic (C16:0) and erucic acids (C22:1) were analyzed, respectively, by GC-MS. Each value and error bar represent average and standard deviation of four biological replicates. The significant difference among labeling abundances is represented by different letters, and was determined with SPSS using univariate Turkey HSD ($p < 0.05$).
